# Some Remarks on Puerperal Sapræmia

**Published:** 1907-10-12

**Authors:** 


### Gynæcology.

### SOME REMARKS ON PUERPERAL SAPRÆMIA.

Of the numerous causes of rises of temperature during the ten days which succeed delivery sapræmia is the commonest. If it were possible to sterilise the vulva midwifery might be carried on as aseptically as ordinary surgery. But until some sufficiently penetrating and non-irritating antiseptic is evolved we can do little more than make the vulva less septic. One looks forward to the day when chemical antiseptics will be relegated to the limbo of the past, and some enterprising physicist will supply us with beneficent rays of sterilisation. Till then it remains impossible to apply forceps without allowing the blades to come into contact with the more or less septic labia, and it is idle to imagine that midwifery can be conducted, even with rubber gloves, upon absolutely aseptic principles, unless no examining finger, no douche-tube, in fact nothing whatever, be allowed to pass through the septic vulva into the vagina. The vulva is, in fact, the weak spot in the whole line of defence against septic infection; but something may be done to strengthen it. Judicious removal of the pubic hair is a proceeding which commends itself to the doctor, if not to the patient. The vulva should be thoroughly washed with ether soap and water before delivery, and again before any operation. After washing, it should be swabbed with lysol, and before any examination is made the labia should be separated, and their inner surfaces swabbed with an antiseptic. In spite of these precautions cases of sapræmia are common. There is another factor in the production of the disease, and that is the retention within the genital tract of suitable pabulum for the growth of micro-organisms. The surface of neglected lacerations becomes sloughy, and harbours immense numbers of organisms; usually the site of their activity is shut off by a protecting barrier of inflammatory exudation, but occasionally the toxins produced in these sloughy lacerations are absorbed, and the symptoms of sapræmia are noticed. The prevention of this form of sapræmia is one of the main indications for the immediate suture of all perineal and labial lacerations. Apart from these, blood-clot, pieces of placenta, shreds of decidua and membrane supply perfect culture media for the growth of saprophytic germs. In the management of the third stage of labour we aim at the absolute elimination of all such remnants from the genital tract.

The symptoms of the disease are moderately definite. The patient does not feel as fit as she ought to do on the third day or so, and she has a continuous frontal headache. She is a little drowsy and dull, but nevertheless does not sleep well. She is generally disinclined for food, and may be constipated. With this there is a gradual rise of temperature and pulse rate, especially in the evening. On physical examination a typical case presents certain very definite signs. The uterus is nearly always tender, and this tenderness does not extend all over the uterus, but is usually localised to that part of the uterine wall upon which the placenta was implanted. If a boss can be felt at this point, and the presence of a fibroid can be excluded, thrombo-phlebitis of the uterine sinuses may be diagnosed. A constant sign is delay in the involution of the uterus. The uterus is, in fact, swollen, just as every other inflamed organ becomes swollen. The involution curve, if properly watched, will show this delay very clearly.

A further characteristic sign of sapræmia is offensiveness of the lochia. The odour may be unmistakable and penetrating; on the other hand it may be faint. The use of pads containing a deodorant is a mistake, because the first evidence of offensiveness is concealed, and it should be remembered that a pad removed from a patient and kept for some hours may very soon become offensive, especially in hot weather.

Subinvolution and its train of misery are the commonest sequelæ of neglected puerperal sapræmia. But there is a graver danger. The organisms which give rise to the disease are too often facultative saprophytes, and there is no assurance that they will not take on a virulent character. There are many cases of puerperal septicæmia which are grafted on cases of sapræmia, and which might have been nipped in the bud by adequate and early treatment.

There are, of course, many mild cases which undergo a natural and spontaneous cure. The treatment of the cases of moderate severity is directed to induce the uterus to expel the offensive material within its cavity. Before undertaking treatment of this nature it is, of course, imperative to make sure that the uterus is at fault, and not the vulva. If there are sloughy lacerations about the vulva and no uterine signs of sapræmia, treat the lacerations with frequent applications of hydrogen peroxide. In cases where the uterus is at fault, try first of all the effect of hot douches, ergot and uterine massage. The massage should be applied through the abdomen while the douche is being given, and the ergot should be given twenty minutes beforehand. In some cases a piece of offensive clot or membrane is expelled, and a cure brought about. But there are others where this fails, and others again in which it is obvious from the first that exploration is necessary. It is best to give an anæsthetic. A vaginal douche is given, and one or two fingers are then inserted through the cervix into the uterine cavity. The cervix will admit two fingers up to the fifth day, and one finger for another five days, so that no dilators are needed. One finger should be used as a rule. The other hand on the abdomen presses the uterus down upon the finger, which can at once appreciate any undue roughness of the uterine cavity. Pieces of adherent placenta or blood-clot can then be removed, and an intra-uterine douche of lysol or iodine water may be given. Now and then brisk hæmorrhage occurs after the removal of a piece of placenta, and it may be necessary to plug the uterus in order to control it. When there is a good deal of offensive material in a soft uterus it is very common to find that a rigor occurs about six hours or so after the exploration, and the friends should be warned that such an occurrence is to be expected.

